# Supplementary material for: Behavioral and emotional difficulties in maltreated children: Associations with epigenetic clock changes and visual attention to social cues
Source: PLoS One. 2025 May 30;20(5):e0321952. doi: 10.1371/journal.pone.0321952 (PMC12124526; doi:10.1371/journal.pone.0321952)
Supplement: Attachment — Submitted filename: Article review.docx [file pone.0321952.s002.docx]

I was asked to review the article entitled “Behavioral and emotional difficulties in maltreated children: Associations with epigenetic clock changes and visual attention to social cues”, submitted to PLOS ONE. The article examines an interesting topic, which is accelerated aging among individuals exposed to child maltreatment. There is currently a research gap (i.e., evidence about telomere length is inconsistent). However, there are some issues that need to be revised before this article can be accepted for publication. Details of my assessment is below.

There is a missing “i” in the word difficulties in the title.

Title: Behavioral and emotional diffculties in maltreated children: Associations with epigenetic clock changes and visual attention to social cues

Abstract: The study design is missing in the abstract and title. This must be explicit in the title or abstract for bibliographic searches and systematic reviews. It’s recommended the authors examine the STROBE guidelines for observational studies <https://www.strobe-statement.org/>

Introduction: The manuscript provided a rationale for assessing epigenetic age acceleration (i.e. deviation of DNA methylation age from chronological age) among children with traumatic experiences. However, the theoretical explanation for assessing eye-tracking (gazing at eyes) as marker for social emotional problems is insufficient. This needs to be expanded, since it’s an independent effect from epigenetic aging. The only information provided was that eye gazing was a behavioral indicator of child maltreatment, but reader is left with uncertainty as to why that’s the case. For instance, children who are victims of maltreatment can suffer eye trauma, which can lead in itself to post-traumatic stress disorder (PTSD). PTSD can cause visual disturbances known as Post-Traumatic Vision Syndrome (PTVS). Alternatively, children who have experienced abuse may learn to avoid eye contact. Also, children may develop social anxiety or suffer from it concurrently. People with social anxiety may avoid eye contact because they perceive it as aversive. In times of extreme trauma, a child may suddenly stare into the distance rather than engage with others (issues in the social engagement system, which is also affected in children with autism, and related to polyvagal theory of trauma). Lastly, people with PTSD may have different pupil responses than those without PTSD. For example, their pupils may not constrict normally when presented with new visual stimuli, or they may dilate more in response to emotional stimuli. These aspects may be already known to the authors, but there are not readily evident to the reader. This reviewer is a pediatric neurologist and specializes in traumatic stress in children.

With regards to objectives, the manuscript presents a central hypothesis which was formally tested. Excellent description.

Methods: Design

As previously noted, the paper should clearly state the type of study design. If the data was collected at a single point in time (snapshot) from any given population (one sample or independent samples are still cross-sectional is the data collection is at single point in time), then the study is cross-sectional. The study would be a case control study if it’s retrospective, meaning the researchers look back in time to identify potential exposures that may have led to a disease. There is no cross-sectional case-control. Perhaps, this is a case control study that have measures collected cross-sectionally and some measures were retrospective. This reviewer does not have enough information to make this determination. If the authors took both epigenetic aging and eye gaze data concurrently but asked other questions about the past that serve as independent predictors, this needs to be clearly stated. Cross-sectional studies can ask questions about the past, but such questions are still collected at a single point in time (cross-sectional). Then, the data are cross-sectional, the study is cross-sectional. However, the authors must indicate which study design they consider in the methodology. Other infrequent study designs could also be nested case control studies if the case control is conducted as part of a prospective study (cohort or a clinical trial). Studies could also be case cohort studies, which are rarer design (In a case-cohort study, cases are defined as those participants of the cohort who developed the disease of interest, but controls are identified before the cases develop. This means that controls are randomly chosen from all cohort participants regardless of whether they have the disease of interest or not, and that baseline data can be collected early in the study). The following work is recommended: Janković, S. (2008). Observational Studies . In: Kirch, W. (eds) Encyclopedia of Public Health. Springer, Dordrecht. https://doi.org/10.1007/978-1-4020-5614-7_2378

The other important aspect is the primary or secondary use of data. If this is an analysis of existing data (regardless if the previous study was conducted by the same authors), then the study is a secondary data analysis. However, if the original study was ongoing and sustained a lapse in time, which was later compensated, then it’s still the same study. Just need to report it as primary data collection and explain the limitations of collecting data in two different times.

With regards to CM and TD group, it’s important to explain who children were recruited into the study. It’s great to know that study protocol was approved by ethics committee. However, there is a need to explain how subjects were protected from retraumatization, if any care was provided (e.g., counseling), and what were some rules to stop trial.

If this was a trial, what was the numbers needed to treat value, if any.

The researchers indicate that the original study did not have a sufficiently large sample size. However, the reader doesn’t have information in the current manuscript about power and sample size calculations and effect size for this study.

The Table 1 is results and should be presented in the Results section, not in the methods.

With regards to variables and measurement, it needs its own subheading in the methods so that the reader can make a distinction between dependent variables (e.g., epigenetic aging and eye gaze), independent variables (CM vs TD), and confounding (i.e., sociodemographics). The manuscript reports a series of instruments with their citation. This is good. However, variables in the study need to be first defined conceptually and then the measure is provided, with details on how the variables were operationalized in the study. The only two variables that were very carefully defined were the dependent variables “Calculation of epigenetic age acceleration” and “gaze patter measurement”. However, independent and confounding were not.

With regards to analysis, serial mediation analysis models were estimated with Lavaan (path analysis). Since the sample size is not very large either in the current study, it’s important to mention of there were adequate power and sample size calculations for the number of parameters in the path analysis. If the authors used latent variables, this needs to be reported. In this regard, the description needs to mention model statistics that were consulted (e.g., likelihood-ratio tests) and the goodness of fit indices that were assessed (e.g., Chi-square for the baseline model vs the estimated, Akaike, and RMSEA) to determine that the model was a good fit to the data.

There is no mention of how missing data was handled. This is very important, particularly for the path analysis (i.e. full information maximum likelihood vs listwise vs other).

Discussion:

The findings suggest an association between CM and the study dependent variables (epigenetic aging and gaze pattern). This association indicate a correlation, not causation, since the data are cross-sectional. Thus, the study needs to indicate this important limitation.

The issue of sample size considerations and power (or underpower) need to be discussed with regards to the results of the path analysis, which did not establish a direct sequential association

between mAge acceleration, visual attention to the eyes, and SDQ scores. Lack of significance may mean the test was underpowered. Power calculations for the path analysis were not provided, so the reader cannot identify if that’s the case.

This reviewer agrees that a study strength is that few studies have formally recognized cases of CM confirmed by child protective services.

With regards to study limitations, if the study is a cross-sectional study or case-control study, it will have different limitations if it’s one or the other. This reviewer is not familiar with a design cross-sectional case-control design. Please provide a citation and why this is the case.

I hope this comments are taken as constructive to improve the manuscript, since the hypothesis tested are very important and the information adds evidence to the body of knowledge albeit the limitations aforementioned.
